# An overview of health workforce education and accreditation in Africa: implications for scaling-up capacity and quality

**DOI:** 10.1186/s12960-022-00735-y

**Published:** 2022-05-07

**Authors:** Sunny C. Okoroafor, Adam Ahmat, James Avoka Asamani, Jean Jacques Salvador Millogo, Jennifer Nyoni

**Affiliations:** Health Workforce Unit, Universal Health Coverage - Life Course Cluster, World Health Organization Regional Office for Africa, Brazzaville, Congo

**Keywords:** Health workforce, Education, Accreditation, Regulation, Universal health coverage, Africa, Health training institutions

## Abstract

**Background:**

For countries to achieve universal health coverage, they need to have well-functioning and resilient health systems. Achieving this requires a sufficient number of qualified health workers and this necessitates the importance of investments in producing and regulating health workers. It is projected that by 2030, Africa would need additional 6.1 million doctors, nurses, and midwives. However, based on the current trajectory, only 3.1 million would be trained and ready for service delivery. To reduce current shortages of the health workforce, Africa needs to educate and train 3.0 million additional health workers by 2030. This study was conducted to describe the distribution and ownership of the health training institutions, production of health workers, and the availability of accreditation mechanisms for training programmes in the WHO African Region.

**Methods:**

A cross-sectional study was conducted using a standardized questionnaire from January 2018 to April 2019. All the 47 countries in the Region were invited to complete a structured questionnaire based on available secondary information from health sector reports, annual HRH reports, country health workforce profiles, and HRH observatories and registries.

**Results:**

Data from 43 countries in the World Health Organization African Region in 2018 show that there were 4001 health training institutions with 410, 1469 and 2122 being medical, health sciences, and nursing and midwifery schools, respectively, and 2221, 1359 and 421 institutions owned by the public, private for-profit and private not-for-profit sectors, respectively. A total of 148 357 health workers were produced in Region with 40% (59, 829) being nurses and midwives, 19% (28, 604) other health workers, and 14% (20 470) physicians. Overall, 31 countries (79%) in the Region have an accreditation framework for the health training institutions and seven countries do not have any accreditation mechanism.

**Conclusion:**

To achieve universal health coverage, matching of competencies with population needs, as well as increasing capacities for health worker production to align with demand (numbers and skill-mix) for improved service delivery should be prioritized, as this would improve the availability of skilled health workforce in the Region.

## Background

One of the United Nations Sustainable Development Goals (SDGs) is to achieve universal health coverage (UHC) by 2030. For countries to achieve this, they need to have well-functioning and resilient health systems to ensure access of the populations to health care services [1]. However, having well-functioning and resilient health systems requires, among other things, a sufficient number of qualified health workers [2]. Hence the challenges of producing them, then the capacity for recruiting, deploying and retaining them, requires sustainable investments [3].

There remains a continual low investment in the education of health workers and a gross mismatch with the disease burden and population needs [4–6]. This has contributed to marked shortages of health workers as well as low absorption of health workers into health systems. Towards achieving UHC, the Global Strategy on Human Resources for Health (HRH) highlights the need for collaboration amongst relevant sectors in ensuring a balance in the health system needs, population needs, education, demand and fiscal space for health workers [7]. Furthermore, investment of the private sector in the education of health workers is essential to boost the production of health workers. Still, there needs to be strong regulatory mechanisms in place to ensure quality training modalities and competence of tutors and trainees [7, 8].

Recent literature has also indicated that poor adherence and enforcement of standards for professional education, licensing, and practice is widespread in the health sector [9–12]. This has been attributed to the global call for an increase in the production and distribution of health workers with efforts geared towards achieving this with quality and professionalism often neglected [10]. This often results in health workers leaning on their discretion in providing services taking into cognizance the levity of professional norms and standards [13]. Often this may result in actions that may not necessarily be beneficial to the patient and population [10].

In 2005, the Africa Region of the World Health Organization (WHO) had only 1.56 million health workers or 3% of the global health workforce stock [14]. Thirteen (13) years on, Africa’s health workforce stock increased to 3.57 million in 2018, representing a 129% increase [14]. However, adjusting to the growth of the population, there was only 61% improvement over 13 years, thus 4.7% real growth in the density of health workers per annum. Despite this increase in the stock of health workers, the density of doctors, nurses, and midwives per 10 000 population in the Region is 15.4 compared to 44.5 needed to attain the median rank of the SDG tracer indicators. Thus, the Africa Region has only 34.6% of the required doctors, nurses, and midwives. It is projected that by 2030, Africa would need additional 6.1 million doctors, nurses, and midwives but in the current trajectory, only 3.1 million would have been trained and ready for service delivery [7, 14]. To reduce current shortages of the health workforce, Africa needs to educate and train 3.0 million additional health workers by 2030 to provide the most essential health services [7].

A scale-up of this magnitude of health workforce will require significant human and financial resources investment to build the human and institutional capacity necessary to produce more health workers and to retain them. The production of health workers in Africa is constrained by limited capacities in terms of shortages of qualified teaching faculty staff, limited teaching and learning materials, as well as inadequate infrastructure and environment for both learning and living for students and lecturers. In addition, the quality of training and the health professionals have continued to be a challenge [15]. There are increasing concerns about the functional accreditation mechanisms in place for health training institutions and programmes. Accreditation of a training institution is the quality control procedure aimed at the official recognition and approval of the training institution or programme after verification of compliance with certain predefined standards by an external body officially designated or recognized for this purpose [16]. On the other hand, retention of health workers is dependent on numerous contextual factors and suggested to require a combination of incentives ranging from enrolment of students of deprived backgrounds, mandatory rural postings, availability of social amenities, enhanced working conditions, etc. [17–20].

The paper presents the distribution and ownership of the health training institutions across the WHO African Region. We also provide insights on the number of health workers produced, and the availability of accreditation mechanisms for training programmes in the Region.

## Methods

A cross-sectional study was conducted using a standardized questionnaire on the distribution and availability of accreditation mechanisms for health training institutions in countries in the WHO African Region from January 2018 to April 2019. All the 47 countries in the Region were invited to complete a structured questionnaire based on available secondary information from health sector reports, annual HRH reports, country health workforce profiles, and HRH observatories and registries. The questionnaire was designed to collect information on the training institutions (faculties, colleges and schools) for the health occupational categories/cadres grouped into medical training institutions (medical practitioners, dentists and pharmacists), nursing and midwifery, and health sciences for other mid-level cadres not captured previously. Ownership of the training institutions (public, private for-profit, and private not-for-profit), the number of graduates produced by the training institutions, and the availability of an accreditation body for the health training institutions were also obtained. Countries were oriented on the questionnaire during a virtual meeting. The questionnaire was completed by the designated staff of the Ministries of Health on behalf of countries and signed off by the Directors/Heads of the HRH department. All questionnaires were completed in English or French by all countries.

The completed questionnaires were reviewed for completeness, data entry and a quality check were done using Epi Info, and analysis was conducted using Microsoft Office Excel. Of the 47 countries in the Region, 43 countries responded giving a response rate of 91%. The countries were Algeria, Angola, Benin, Botswana, Burkina Faso, Burundi, Cape-Verde, Central African Republic, Chad, Congo, Cote d'Ivoire, Democratic Republic of Congo, Eritrea, Eswatini, Ethiopia, Gabon, Gambia, Ghana, Guinea, Guinea-Bissau, Kenya, Lesotho, Liberia, Madagascar, Malawi, Mali, Mauritania, Mauritius, Mozambique, Namibia, Niger, Nigeria, Rwanda, Senegal, Seychelles, Sierra Leone, South Africa, South Sudan, Tanzania, Togo, Uganda, Zambia and Zimbabwe. Cameroun, Comoros, Equatorial Guinea and Sao Tome and Principe did not complete the survey.

## Results

### Health training institutions by country

Overall, there were 4001 health training institutions in the 43 countries that responded as shown in Table 1. One thousand and twenty-eight (26%) of the institutions were located in Nigeria, 870 (22%) in the Democratic Republic of Congo, and 281 (7%) in Tanzania. Four hundred and ten (410) of the 4001 health training institutions (10%) were medical training institutions with the Democratic Republic of Congo, Nigeria and Ethiopia having a total of 190 (46%) of the 410 medical training institutions in the Region (Democratic Republic of Congo—102 (25%), Nigeria—52 (13%) and Ethiopia—36 (36%)). The health sciences training institutions were 1,469 (37% of the health training institutions) with 1104 (75%) of the 1469 health sciences training institutions are located in four (4) countries: Nigeria—714 (49%), Tanzania—185 (13%), Democratic Republic of Congo—110 (7%) and Angola—95 (6%)). Fifty-three percent (2122) of the health training institutions in the Region are for training nurses and midwives. Of this number, 50% (1054) are in the Democratic Republic of Congo (658 representing 31%), Nigeria (262 representing 12%) and Chad (134 representing 6%).

**Table 1:** Distribution of health training institutions by country

Country name	Medical training institutions	Health sciences institutions	Nursing and midwifery institutions	Total
Algeria	15	3	113	131
Angola	18	95	8	121
Benin	2	10	2	14
Botswana	2	3	7	12
Burkina Faso	4	48	51	103
Burundi	4	25	19	48
Cape-Verde	5	8	5	18
Central African Republic	1	6	1	8
Chad	2	3	134	139
Congo	2	1	6	9
Cote d'Ivoire	4	0	1	5
Democratic Republic of Congo	102	110	658	870
Eritrea*	1	–	–	1
Eswatini	–	3	4	7
Ethiopia	36	20	40	96
Gabon	1	1	2	4
Gambia	1	2	4	7
Ghana	13	23	103	139
Guinea	3	38	42	83
Guinea-Bissau	2	2	3	7
Kenya*	25	–	–	25
Lesotho	–	2	6	8
Liberia	1	8	19	28
Madagascar	7	2	94	103
Malawi	4	4	9	17
Mali	5	–	80	85
Mauritania	1	1	4	6
Mauritius	2	1	1	4
Mozambique	15	50	34	99
Namibia	1	11	12	24
Niger	3	6	37	46
Nigeria	52	714	262	1028
Rwanda	1	2	5	8
Senegal	5	3	55	63
Seychelles	–	1	1	2
Sierra Leone	1	–	–	1
South Africa*	28	–	–	28
South Sudan	3	26	26	55
Tanzania	12	185	84	281
Togo	2	4	14	20
Uganda	12	–	71	83
Zambia	8	36	72	116
Zimbabwe	4	12	33	49
Total	410	1469	2122	4001

^*^Institutions in these countries provided training programmes for all health occupational categories/cadres

### Ownership of training institutions

Table 2 shows that of the 4001 health training institutions in the Region, 2221 (56%) were owned by the public sector, and 44% by the private sector 1359 (34%) by the private for-profit sector and 421 (10%) by private not-for-profit sector). In addition, 53% (2122) of the institutions were nursing and midwifery institutions, 37% (1469) were health sciences institutions, and 10% (410) were medical training institutions. 64% (264) of the 410 medical training institutions were owned by the public sector, 27% (111) by the private for-profit sector, and 9% (35) by the private not-for-profit. For the health sciences institutions, 69% (1015), 25% (367) and 6% (87) are owned by the public, private for-profit and private not-for-profit sectors, respectively.

**Table 2:** Distribution of health training institutions by sector

Training institutions	Public sector		Private for-profit sector		Private not-for-profit sector		Total	
	Number (%)		Number (%)		Number (%)		Number (%)	
Medical training institutions	264	64%	111	27%	35	9%	410	10%
Health sciences institutions	1015	69%	367	25%	87	6%	1469	37%
Nursing and midwifery institutions	942	44%	881	42%	299	14%	2122	53%
Total	2221	56%	1359	34%	421	10%	4001	100%

### Production of health workers

**Fig. 1:**
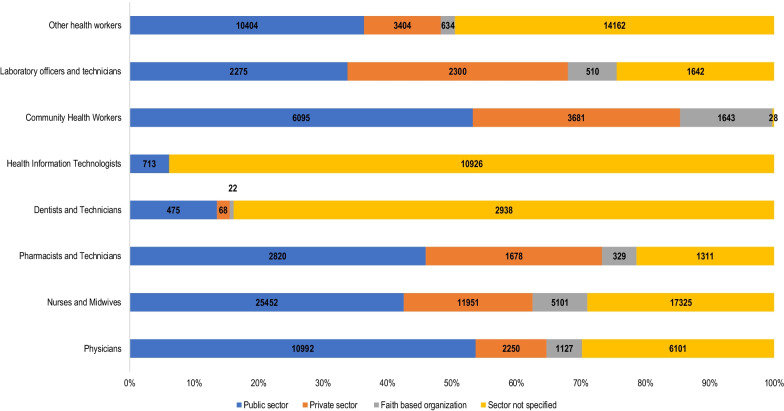
Annual graduates in the African Region in 2018

A total of 148 357 health workers were produced in the Africa Region in 2018 (Fig. 1). Of this 40% (59, 829) were nurses and midwives, 19% (28, 604) other health workers, and 14% (20 470) physicians. Health information technologists (11 639) and community health workers (11 447) comprised 8%, and laboratory officers and technicians, and dentists and technicians comprised 5% (6727) and 4% (6138), respectively. Forty percent of all health workers were produced by public sector-owned health institutions (*n* = 59 226) with 54% (*n* = 10 992) of physicians, 53% (*n* = 6025) community health workers, 46% (*n* = 2860) pharmacists and technicians and 43% (25 452) nurses and midwives graduating from public sector-owned schools.

### Accreditation mechanisms for training institutions

**Fig. 2:**
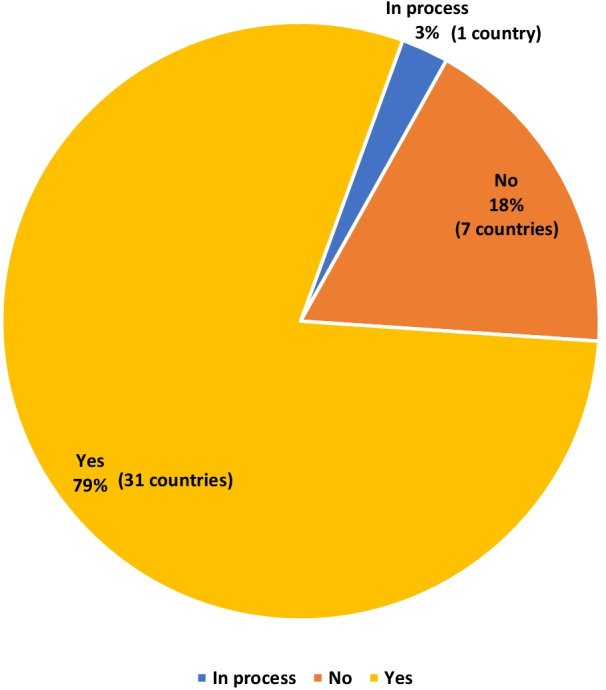
Percentage of countries with an accreditation body for health training institutions in the African Region (*N* = 39)

To ascertain the progress made by countries towards strengthening the capacity and quality of educational institutions through their accreditation of programmes in their training schools, countries were asked to provide information on the availability of a national accreditation mechanism for health training institutions. Figure 2 shows the percentage of countries with accreditation mechanisms in place of the 39 countries that provided information. Thirty-one (31) countries (79%) had an accreditation framework for health training institutions. The countries with accreditation mechanisms were Angola, Botswana, Burundi, Cape-Verde, Central African Republic, Cote d'Ivoire, Democratic Republic of Congo, Eritrea, Eswatini, Ethiopia, Gabon, Ghana, Guinea, Guinea-Bissau, Lesotho, Liberia, Madagascar, Malawi, Mauritius, Mozambique, Namibia, Niger, Nigeria, Rwanda, Senegal, Seychelles, Sierra Leone, Tanzania, Uganda, Zambia and Zimbabwe. Algeria, Benin, Burkina Faso, Congo, Mali, Mauritania and Togo (18% of the countries) do not have an accreditation mechanism. Chad was in the process of establishing an accreditation mechanism at the time of the study.

## Discussion

For countries to achieve UHC, they need to invest in ensuring the availability of qualified, skilled, and motivated health workers [21, 22] and this can be achieved by increasing production, retention and regulation capacities [9, 18]. Concerted efforts should aim at increasing the numbers, distribution and production capacity in the Region to produce 3 million additional health workers by 2030 and this should be led by countries. The evidence on the health systems and population needs should be matched to supply and demand for health workers [7, 22]. To achieve this, leadership, coordination and planning for the health workforce should be strengthened. Investment in pre-service education applying an intersectoral approach with relevant ministries and parastatals (health, education, finance, planning and budgeting) should be scaled–up [7, 23]. Matching competencies with population needs, as well as increasing capacities for health worker production and recruitment to align with demand (numbers and skill-mix) for service delivery, will improve the availability of skilled health workforce needed to achieve UHC. Additionally, harmonization of curricula, education standards, accreditation, and professional regulation across all levels and sectors, as well as promoting career progression and retention of tutors will ultimately ensure that a fit-for-purpose health workforce is available to deliver integrated health services and improve the health indices in the Region [22, 24].

Our findings indicate that the private sector was actively investing in health workforce education and thereby contributing to increasing the production capacity in the Region. Whilst this is laudable and should be encouraged, countries need to ensure policies are in place to encourage potential investors and ensure alignment of quality standards in the public and private sectors [7, 8]. These policies should focus on ensuring needed infrastructure for quality training is in place, enrolment processes are based on gender and other equity considerations, the processes of selecting students consider socio-demographic factors to ensure retention after graduation, and tutors have relevant experience and qualifications [7, 25].

In addition, countries need to ensure that efforts aimed at increasing the production capacity should be complemented with strong governance and regulatory frameworks to ensure that quality and accountability is ensured [10, 21]. This is pertinent as weak HRH regulation and non-participation of regulatory bodies in HRH planning and development are evident in some countries. Additionally, variations in technical competencies, skills and professionalism amongst qualified health workers have been reported due to varied standards of education and poor regulation [10, 11, 26]. This has resulted in fragmentation of HRH regulation and practice and the weakening of the regulatory bodies and processes. Furthermore, the non-alignment of global, regional and national policy reforms with professional regulation and practice have been impeding service delivery and the achievement of health sector goals at various levels. Therefore, there is a need to foster the active participation of professional regulatory bodies in HRH planning and development. The establishment or strengthening of professional regulatory bodies to enforce laws and regulations including HRH accreditation, systems for licensure, professional development, disciplinary measures, education standards, quality assurance, codes of conduct and scopes of practice [25, 27] will promote ethical practices and accountability. Harmonizing the practices and regulations of professional regulatory bodies between professions and across all levels will also foster inter-professional collaboration and reduce rivalries. Ultimately, functional regulatory mechanisms will ensure the availability of qualified, skilled, responsive and productive health workers, quality of care and a well-performing health system [28]. By ensuring adequate regulation, optimal standards of care will be provided, the population will be protected from harm, health workers would be accountable as incompetence and negligence would be at the barest minimum, and inefficiencies would be eliminated [28, 29].

## Conclusion

The Africa Region is faced with shortages of health workers, and low production and regulatory capacities. This is compounded by a prolonged low investment in the education of health workers and a gross mismatch with the disease burden and population needs. To close the projected shortfall of 6.1 million doctors, nurses, and midwives by 2030, the countries in the Region need to increase the production and distribution of health workers, and this should be complemented by establishing/strengthening and enforcing the standards for professional education, licensing, and practice. This would ultimately improve the availability of skilled health workforce needed to achieve universal health coverage.

## Data Availability

The data for the findings are available on request from the health workforce unit of WHO AFRO.
